# MoESurv: a zero-sample and transferable survival prediction framework for rare cancers using mixture of experts

**DOI:** 10.1093/bioinformatics/btag549

**Published:** 2026-07-22

**Authors:** Shuping Fang, Yuhang Wang, Mengyan Zhou, Yanting Shao, Zhenghao Jiang, Yitong Guo, Tao Jiang, Feng Wang, Jingya Fang, Hong Tian

**Affiliations:** Jiangsu Key Laboratory of Druggability of Biopharmaceuticals and State Key Laboratory of Natural Medicines, School of Life Science and Technology, China Pharmaceutical University Nanjing, Nanjing, Jiangsu, P.R. China; Regulatory Science, Jiangsu Simcere Pharmaceutical Co Ltd, Nanjing, Jiangsu, P.R. China; State Key Laboratory of Neurology and Oncology Drug Development, Jiangsu Simcere Pharmaceutical Co Ltd, Nanjing, Jiangsu, P.R. China; School of Science, China Pharmaceutical University, Nanjing, Jiangsu 211198, P.R. China; Jiangsu Key Laboratory of Druggability of Biopharmaceuticals and State Key Laboratory of Natural Medicines, School of Life Science and Technology, China Pharmaceutical University Nanjing, Nanjing, Jiangsu, P.R. China; State Key Laboratory of Neurology and Oncology Drug Development, Jiangsu Simcere Pharmaceutical Co Ltd, Nanjing, Jiangsu, P.R. China; School of Life Science and Technology, China Pharmaceutical University, Nanjing, Jiangsu 211198, P.R. China; School of Life Science and Technology, China Pharmaceutical University, Nanjing, Jiangsu 211198, P.R. China; Mudi Meng Honors College, China Pharmaceutical University, Nanjing, Jiangsu 211198, P.R. China; Research Institute of General Surgery, Jinling Hospital, Affiliated Hospital of Medical School, Nanjing University, Nanjing, Jiangsu, P.R. China; State Key Laboratory of Neurology and Oncology Drug Development, Jiangsu Simcere Pharmaceutical Co Ltd, Nanjing, Jiangsu, P.R. China; School of Science, China Pharmaceutical University, Nanjing, Jiangsu 211198, P.R. China; Jiangsu Key Laboratory of Druggability of Biopharmaceuticals and State Key Laboratory of Natural Medicines, School of Life Science and Technology, China Pharmaceutical University Nanjing, Nanjing, Jiangsu, P.R. China

## Abstract

**Motivation:**

Accurate survival prediction is crucial for personalized cancer treatment but remains challenging for rare cancers due to limited data. Most deep learning models require large training datasets, which are unavailable for rare cancer types, creating a significant clini-cal bottleneck.

**Results:**

We propose MoESurv, a zero-sample survival prediction framework that leverages a mix-ture-of-experts architecture to extract generalizable prognostic patterns from pan-cancer data. The model integrates shared experts, cancer-specific experts, and routing experts within an autoencoder to disentangle common and type-specific survival features. Evaluated on seven rare TCGA cancer types, MoESurv achieved state-of-the-art performance, improving the average C-index by 4 percentage points over the best baseline. Further-more, external validation across diverse populations and independent cohorts—including a Chinese glioma cohort (CGGA mRNAseq_693, C-index = 0.7433), a rare GBM IDH-mutant subtype (C-index = 0.8064), and the pan-cancer PCAWG cohort (C-index = 0.7090)—demonstrated that MoESurv possesses the most robust predictive per-formance, highlighting its generalizability. MoESurv also effectively stratified high- and low-risk patient groups and identified potential survival-associated genes, demonstrating both clinical utility and biological interpretability.

**Availability:**

The code is freely available at https://github.com/HuaYC666/MoESurv and https://zenodo.org/records/20785500.

## 1 Introduction

Accurate prognosis assessment is critical for personalizing treatment strategies and improving clinical outcomes in cancer patients (Cooperberg *et al*. 2023). In recent years, deep learning-based survival prediction models leveraging high-throughput transcriptomic data have made significant progress (Morganti *et al*. 2019). These models can capture complex molecular patterns closely associated with patient survival, enabling more precise risk stratification and outcome prediction, which in turn assists clinicians in optimizing treatment plans (Moon *et al*. 2023, Grist *et al*. 2025). Moreover, by linking gene expression with clinical endpoints, researchers can identify risk and protective genes with independent prognostic value (Gao *et al*. 2013, Akcakanat *et al*. 2021), offering important clues for uncovering disease mechanisms and developing targeted therapies.

However, the development of most existing survival prediction models heavily relies on large-scale, high-quality training datasets. While feasible for common cancers such as lung or breast cancer, this requirement poses a major challenge for rare cancers—including adrenocortical carcinoma (ACC) and pheochromocytoma/paraganglioma (PCPG)—due to their very low incidence (Payabyab *et al*. 2016, Carrasquillo *et al*. 2021). The limited availability of samples hinders the construction of robust and accurate predictive models, leaving clinicians without reliable decision support for risk stratification and management. This represents a key obstacle in the field of precision medicine.

An intuitive hypothesis is that, despite etiological and pathological heterogeneity across cancer types, they may share conserved biological processes—such as cell proliferation, immune evasion, or metabolic reprogramming—that are relevant to disease progression and prognosis. If such generalizable “shared prognostic information” could be effectively extracted from data-rich common cancers and transferred to rare cancers, it might be possible to overcome sample size limitations and achieve accurate survival risk prediction in a zero-sample setting.

Motivated by this, we propose a cross-cancer zero-sample survival prediction framework named MoESurv (Mixture of Experts for Survival Analysis). The model deeply integrates a mixture-of-experts architecture with an autoencoder, incorporating shared experts, routing-experts, cancer-specific experts, and a routing network that work cooperatively. Shared experts extract generalizable prognostic features across cancers, specific experts handle cancer-type heterogeneity, routing experts are designed to extract heterogeneity not attributable to cancer type (such as intra-cancer heterogeneity), and the routing network dynamically assigns samples to appropriate routing experts. This design enables the identification of multiple types of heterogeneity while more efficiently capturing features shared across cancer types.

In evaluations across seven rare or small-sample cancer types, MoESurv achieved the best overall zero-sample survival prediction performance, improving the average C-index by 4 percentage points over the best baseline, with a 6-point lead in KICH. Furthermore, the model helped identify potential survival-associated genes in rare cancers such as KICH and MESO, offering clues for mechanistic studies and targeted therapy development. MoESurv provides a effective solution to the problem of missing prognostic models for rare cancers, with both clinical and scientific relevance. Furthermore, external validation across diverse populations and independent cohorts—including a Chinese glioma cohort (CGGA mRNAseq_693, C-index = 0.7433), a rare GBM IDH-mutant subtype (C-index = 0.8064), and the pan-cancer PCAWG cohort (C-index = 0.7090)—demonstrated that MoESurv possesses the most robust predictive performance, highlighting its generalizability.

## 2 Related work

Survival analysis methods can be broadly categorized into three groups: parametric models, semi parametric models represented by the Cox proportional hazards (CoxPH) model, and discrete time interval based models. Parametric models require assuming that patient survival times follow a specific distribution (Martinsson 2016), such as exponential or Weibull. However, this assumption can constrain model learning, especially when the actual data distribution deviates from the presumed form, potentially leading to significant performance degradation. To overcome this limitation, the CoxPH model assumes only proportional hazards for covariates while leaving the baseline hazard unspecified. By maximizing the partial likelihood to estimate covariate effects without imposing a parametric form on the baseline hazard, the CoxPH model maintains flexibility and enables robust analysis of survival data. Discrete time models divide the time axis into intervals and estimate survival probabilities within each interval (Lee *et al*. 2018, Zhao and Feng 2020), which allows them to operate without relying on the proportional hazards assumption. Although both parametric and discrete time models possess certain advantages, their implementation is often more cumbersome, making the CoxPH model the most widely adopted approach in practice.

Deep learning methods, with their adaptive fitting capacity, have been increasingly applied to survival analysis using transcriptomic data, often within the CoxPH framework. For instance, Cox nnet (Ching *et al*. 2018) uses neural networks to extract hidden features for Cox based modeling. Similarly, DeepSurv (Katzman *et al*. 2018) employs neural networks to predict patient prognosis and recommend personalized treatment strategies via hazard ratios. To incorporate prior biological knowledge, DeepOmics (Zhao *et al*. 2021) introduces gene pathway information through masked input layers, enabling pathway aware feature extraction. Another approach transforms gene expression profiles into image like representations and applies convolutional neural networks for feature learning and risk prediction (Yan *et al*. 2025). Graph based methods such as AGGSurv (Ling *et al*. 2022) leverage multi view sparse graphs to capture comprehensive relationships in gene expression data.

Most supervised learning methods rely on large training datasets to achieve good performance. However, biological data are often limited in sample size, making small sample or zero sample survival prediction an active research direction. Denoising autoencoders (Yousefi *et al*. 2017) have been used to extract pan cancer representations from transcriptomic data for survival prediction. Building on this, VAECox (Kim *et al*. 2020) employs a variational autoencoder to capture the latent distribution of gene expression and performs CoxPH modeling in that space, improving prediction in small sample settings. Methods like AUTOSurv (Jiang *et al*. 2024) integrate pathway information with variational autoencoders to mitigate overfitting in high dimensional, samll sample scenarios, achieving strong performance in ovarian and breast cancer prognosis. Federated learning techniques, illustrated by MOSAHit (Wen and Li 2025), enable knowledge transfer across institutions under data scarce conditions. Other small sample strategies include SVM based models tailored for predicting 5 year overall survival in hepatocellular carcinoma (Jiang *et al*. 2025), and meta learning frameworks (Qiu *et al*. 2020) that train a transferable feature extractor on pan cancer data, which can be quickly adapted to new cancer types with minimal fine tuning. Extensions of meta-learning method into multi omics data have also been proposed, such as MMOSurv (Wen and Li 2025).

While small sample learning approaches partially address the challenge of rare cancers, they still require at least some target domain samples and therefore do not constitute a strict zero sample solution. At the same time, the selection of the small-size examples provided to the model for fine-tuning often significantly influences the model’s predictive performance. For rare cancers where even small sample data are unavailable or hard to choose, developing genuine zero sample prediction methods remains an important unmet need.

## 3 Methods

### 3.1 Datasets and preprocessing

Gene expression data used in this study were obtained from The Cancer Genome Atlas (TCGA) database (Tomczak *et al*. 2015). To evaluate the zero-sample prediction performance of MoESurv on rare cancer types, gene expression data from 18 common cancer types (Table 1) were used for model training, while data from seven relatively rare or small-sample-size cancer types in TCGA (Table 2)—such as READ—were reserved for testing. All datasets were normalized during preprocessing using transcripts per million (TPM) transformation to mitigate biases arising from gene length and sequencing depth. Following TPM normalization, a log(*x* + 1) transformation was applied to reduce the influence of extreme values and approximate a more Gaussian distribution of the data.

**Table 1:** Summary of datasets used in the unsupervised training of MoESurv.

Cancer types	Full name	Patients	Prop.Censored
BRCA	Breast Cancer	1210	0.8364
KIRC	Kidney Clear Cell Carcinoma	603	0.6683
LUAD	Lung Adenocarcinoma	565	0.6301
HNSC	Head and Neck Cancer	563	0.5506
LGG	Lower Grade Glioma	521	0.7505
SKCM	Melanoma	455	0.5297
STAD	Stomach Cancer	443	0.6230
OV	Ovarian Cancer	425	0.3765
BLCA	Bladder Cancer	425	0.5576
LIHC	Liver Cancer	420	0.6143
SARC	Sarcoma	264	0.6250
KIRP	Kidney Papillary Cell Carcinoma	320	0.8406
CESC	Cervical Cancer	309	0.7638
UCEC	Endometrioid Cancer	193	0.8135
PAAD	Pancreatic Cancer	183	0.4809
LAML	Acute Myeloid Leukemia	161	0.3540
GBM	Glioblastoma	165	0.2000
ESCA	Esophageal Cancer	195	0.5846

**Table 2:** Summary of dataset from rare cancer types or small-sample-size cancer types pending survival prediction.

Cancer types	Full name	Patients	Prop.Censored
ACC	Adrenocortical Cancer	77	0.6494
CHOL	Bile Duct Cancer	45	0.4889
READ	Rectal Cancer	102	0.8039
KICH	Kidney Chromophobe	89	0.8652
MESO	Mesothelioma	86	0.1512
PCPG	Pheochromocytoma & Paraganglioma	185	0.9568
UCS	Uterine Carcinosarcoma	57	0.3860

For external validation, we used data from the PCAWG (Underwood 2020) and CGGA (Zhao *et al*. 2021) databases. The PCAWG pan-cancer cohort was used to assess the generalizability of MoESurv across different patient cohorts. The CGGA mRNAseq_693 glioma cohort, including its rare GBM IDH-mutant subset, was employed to evaluate the model’s performance across ethnic groups and its applicability to rare cancer types. We first identified the overlapping genes between the TCGA dataset and each external cohort. Gene expression matrices were then log-transformed using log(TPM + 1). The preprocessed TCGA data, encompassing 18 cancer types, was used to train the model, which was subsequently tested directly on the held-out external cohorts.

### 3.2 Cancer-type-specific mixture-of-experts system

The Mixture of Experts (MoE) is an advanced machine learning framework that uses a routing mechanism to selectively activate specialized expert models based on input characteristics. While MoE has been widely applied in natural language processing (Liu *et al*. 2024, Zhu *et al*. 2025), its potential remains largely unexplored in biological data analysis.

Cancer is highly heterogeneous, with substantial differences in gene expression patterns not only between cancer types but also within the same cancer type (Dentro *et al*. 2021). To build a generalizable survival prediction model, it is essential to capture common prognostic features across cancers while appropriately filtering out inter- and intra-type heterogeneity. We therefore introduce MoESurv, which employs a mixture of three expert modules with distinct roles. Shared experts extract universal gene-expression features across cancers, and cancer-type experts model type-specific characteristics. Crucially, the routing experts act as a specialized filtering system. A gating network evaluates each input sample and dynamically routes it to the most appropriate routing expert, which has become specialized in processing a particular type of genomic pattern during training. This cooperative mechanism ensures a balanced and comprehensive analysis of both shared and idiosyncratic signals. Each expert is implemented as a feed-forward network with a hidden dimension of l=1024. The network first projects the input gene expression matrix from its original dimension to an intermediate 2048-dimensional space. After applying the ReLU non-linearity (Agarap 2018), the features are mapped to the 1024-dimensional latent space, which serves as the expert’s output representation. Suppose there are $n_{s}$ shared experts, $n_{r}$ routing experts, and for each cancer-type, there are $n_{c}$ cancer experts. Let $x_{i}\in R^{g}$ denote the gene expression vector of the *i*-th sample, where g is the number of genes. MoESurv performs initial feature extraction from gene expression data using its expert modules:


(1) h(s)=∑k=1nsFFNk(s)(xi),h(c)=∑k=1ncFFNk,j(c)(xi),h(r)=∑k=1nrgi,tFFNk(r)(xi),FNN(xi)=W2·ReLU(W1xi+b1)+b2, W1∈R2048×g,W2∈Rl×2048ReLU(x)={x, x>00,otherwise, 

Where $h^{\left(s\right)},h^{\left(c\right)},h^{\left(r\right)}\in R^{l}$. ${\mathrm{FFN}}_{k}^{\left(s\right)}$ corresponds to the *k*-th shared expert, ${\mathrm{FFN}}_{k,j}^{\left(c\right)}$ denotes the *k*-th cancer expert for the *j*-th cancer type, and ${\mathrm{FFN}}_{k}^{\left(r\right)}$ refers to the *k*-th routing expert. The weight $g_{k,i}$ assigned to the *k*-th routing expert for the i-th sample is computed to adaptively select the most suitable routing experts for each sample, as follows:


(2) gk,i=gk,i′∑j=1nrgj,i',gk,i'={sk,i,sk,i∈Topk({sj,i|1≤j≤nr},Kr),0,otherwisesk,i=Sigmoid(xiTek),

Where $s_{k,i}$ denotes the score assigned by the router to the *k*-th expert for the *i*-th sample. The router acts as a gating mechanism that performs binary logistic regression on the input data to determine the suitability of each routing expert. $e_{k}\in R^{g}$ represents the regression coefficient vector used to compute the gating score for the k-th routing expert. A value of $s_{k,i}$ closer to 1 indicates that the corresponding expert is more suitable for feature extraction from the current sample. The function $\mathrm{Topk}(\cdot ,K_{r})$ selects the top $K_{r}$ (defaults to 1) experts with the highest scores for each sample, while setting the scores of all other experts to zero. To ensure balanced utilization of all routing experts and prevent the model from repeatedly activating only a fixed subset, a load-balancing loss $L_{b}$ is introduced following prior work (Liu *et al*. 2024). This loss encourages all experts to be adequately trained during the learning process:


(3) $$L_{b}=n_{r}\sum_{i=1}^{n_{r}}{\bar{g}}_{\mathrm{batch},i}P_{\mathrm{batch},i},$$

Where ${\bar{g}}_{\mathrm{batch},i}$ is the average gating weight of the *i*-th routing expert over the batch of samples that input the router, and $P_{\mathrm{batch},i}$ denotes the empirical probability of the expert being selected within the same batch of samples.

### 3.3 Mixture autoencoder

Unlike traditional mixture-of-experts systems, MoESurv does not directly combine features from different expert modules through summation and then excute supervised training. Instead, it further employs these features to construct a mixture autoencoder. An autoencoder is a deep learning-based model that maps high-dimensional data into a low-dimensional space, learning latent representations of the data. It has been widely applied for dimensionality reduction and feature extraction in gene expression data analysis (Li *et al*. 2023, Qiu *et al*. 2025). MoESurv introduces the concept of a mixture autoencoder to extract generalizable features across multiple cancer types. The model first computes latent feature representations from each expert module independently, then combines these feature vectors via element-wise summation:


(4) $$h_{i}^{\left(m\right)}=h_{i}^{\left(s\right)}+h_{i}^{\left(c\right)}+h_{i}^{\left(r\right)},\;h_{i}^{\left(m\right)}\in R^{l},$$

Where $h_{i}^{\left(s\right)}$, $h_{i}^{\left(c\right)}$ and $h_{i}^{\left(r\right)}$ denote the feature vectors extracted by the shared expert, cancer expert, and routing expert modules, respectively. To ensure that the mixture latent features are compact and effectively represent the gene expression data, MoESurv adopts an asymmetric decoder structure. During the reconstruction phase, the decoder performs two linear transformations. First, the mixture latent features of dimension l are projected to a 2048-dimensional latent space; subsequently, they are mapped back to the original input dimension of gene expression. This process is formulated as:


(5) $${\hat{x}}_{i}=W_{2}\cdot \mathrm{ReLU}(W_{1}h_{i}^{\left(m\right)}+b_{1})+b_{2},$$

Where $h_{i}^{\left(m\right)}\in R^{l}$ denotes the vector of mixture latent features. $W_{1}$ and $W_{2}$ are the linear-transform matrices, respectively, and $b_{1}$, $b_{2}$ are the corresponding biases. The model is trained by minimizing the mean squared error reconstruction loss:


(6) $$L_{\mathrm{recon}}=\frac{1}{n}\sum_{i=1}^{n}{{\left||x_{i}-{\hat{x}}_{i}|\right|}_{2}}^{\;}.$$

This loss function guides the network to learn low-dimensional embeddings that summarize the input data effectively, encouraging the expert modules to collaboratively extract features of different cancer types.

### 3.4 Generalizable supervised training paradigm

**Figure 1:**
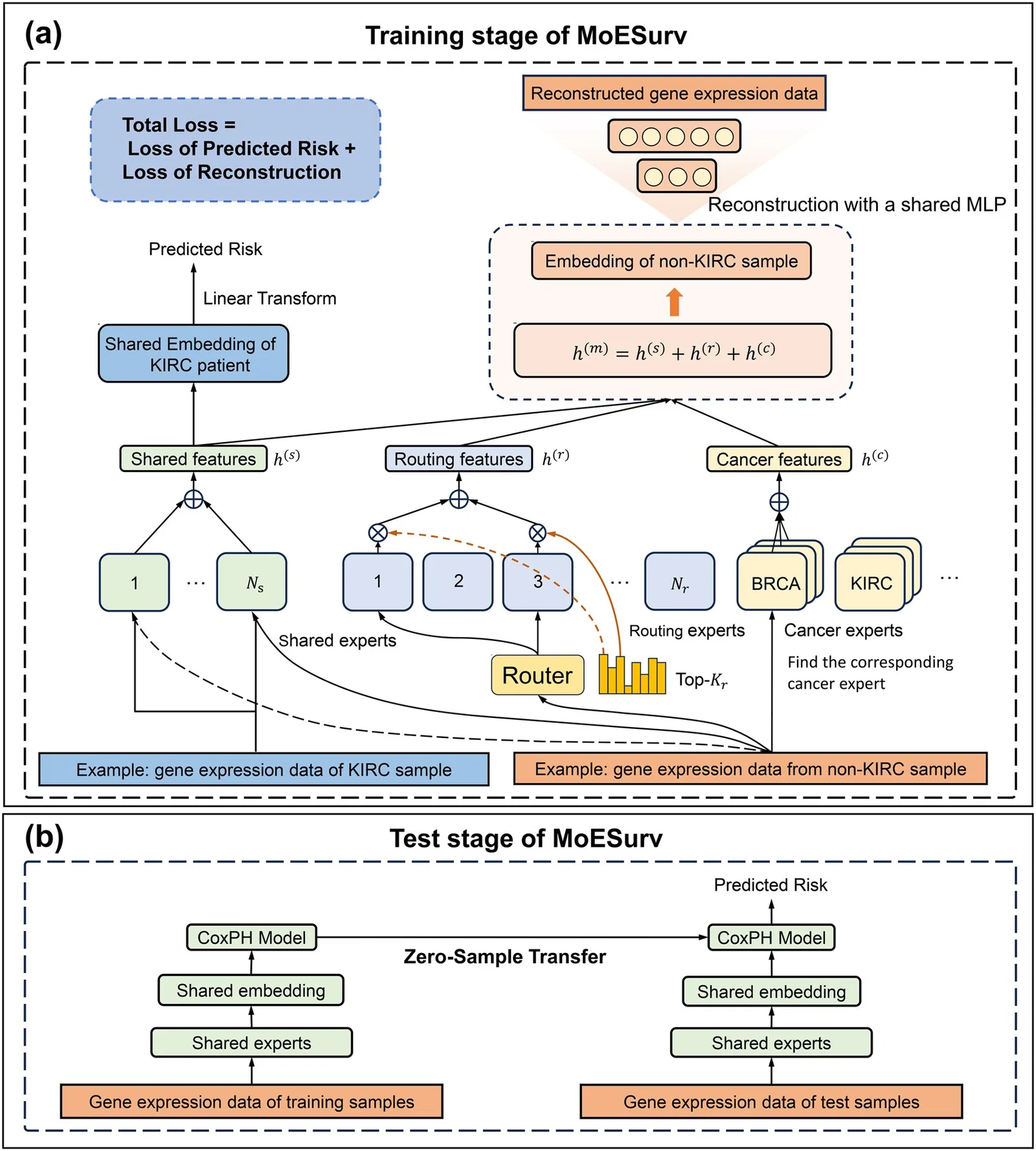
The MoESurv framework. (a) Training process of MoESurv. The model employs three types of experts to learn latent representations from gene expression data: shared experts, which extract features common to all cancer types; routing experts extract features for cancer samples with specific patterns based on the router’s selection; and cancer-type experts, which specialize in individual cancer types. The outputs of these experts are combined via element-wise summation to form a mixture representation, which is then passed to a shared decoder to reconstruct the original gene expression profile. In addition to the reconstruction task, MoESurv incorporates a survival prediction loss computed from the shared features of cancers not used in the reconstruction step. This encourages the shared experts to learn generalizable survival-related features rather than focusing solely on reconstruction. (b) Zero-shot prediction process of MoESurv. During testing, a CoxPH model is first trained on the shared features derived from all non-target cancer types. This risk prediction model is then directly applied to the shared features of the target rare cancer samples, enabling survival prediction without any target-specific training.

Although the mixture autoencoder enables expert modules to focus on learning their respective feature types, thereby helping the shared expert module capture common expression patterns in cancer and extract generalizable features, this approach alone is insufficient. The commonly shared features may not necessarily be strongly associated with patient survival, potentially introducing significant noise and compromising the generalization capability of the shared representations. We designed a generalizable supervised training paradigm to enhance the model’s ability to extract not only features useful for reconstruction but, more importantly, survival-related features that generalize across different cancer types (Fig. 1). Let $I_{i}$ denote the time until the event of interest (death) for the i-th sample, and $C_{i}$ the censoring time. The observed time is $O_{i}=\min(I_{i},C_{i})$, and $\delta_{i}=I(I_{i}<C_{i})$ indicates whether the event was observed. In each iteration, one cancer type is randomly selected as the survival prediction task, while the remaining 17 types are used for reconstruction. Two batches are then sampled: a survival batch $B_{\mathrm{risk}}$ of size $n_{\mathrm{risk}}$(defaults to 256) from the selected cancer type, and a reconstruction batch $B_{\mathrm{recon}}$ of size $n_{m}$ (defaults to 512) from the remaining cancer types. To account for limited sample sizes in individual cancers, $B_{\mathrm{risk}}$ is sampled with replacement, whereas $B_{\mathrm{recon}}$ is sampled without replacement. The reconstruction loss is computed as:


(7) $$L_{\mathrm{recon}}=\frac{1}{n_{m}}\sum_{i=1}^{n_{m}}{{\left||x_{i}-{\hat{x}}_{i}|\right|}_{2}}^{\;},x_{i}\in B_{\mathrm{recon}}$$

Where ${\hat{x}}_{i}$ is the output of the mixture autoencoder. To guide the shared experts toward survival-related patterns rather than mere expression reconstruction, we employ the negative log partial likelihood loss, widely used in Cox proportional hazards models:


(8) $$L_{\mathrm{risk}}=-\sum_{i=1}^{n_{\mathrm{risk}}}\delta_{i}[\beta^{T}h_{i}^{\left(s\right)}-\log\sum_{j:{O_{j}>O}_{i}}\exp\left(\beta^{T}h_{j}^{\left(s\right)}\right)\;],\;$$

Where $h_{i}^{\left(s\right)}$ and $h_{j}^{\left(s\right)}$ are features from $B_{\mathrm{risk}}$ extracted by the shared experts, and β is a learnable coefficient vector. Since the routing experts are not involved in survival prediction, the load-balancing loss $L_{b}\;$is only applied during reconstruction. The total loss is defined as:


(9) $$L_{\mathrm{total}}=\gamma_{1}L_{\mathrm{recon}}+\gamma_{2}L_{\mathrm{risk}}+\gamma_{3}L_{b},$$

With $\gamma_{1}=\gamma_{2}=1$. Following prior work (Zhu *et al*. 2025), we set $\gamma_{3}=0.01$ to encourage balanced usage of routing experts without imposing a strong constraint. The model is optimized for 3000 iterations using the Adam optimizer with a learning rate of 1 × 10 − 5.

The model performs zero-sample survival prediction on rare cancers by leveraging shared features identified across 18 cancer types. A CoxPH model, fitted on these features and time-to-event data from training data, subsequently predicts risk scores for a rare cancer based on its own shared features extracted by shared expert trained in training data.

### 3.5 Evaluation metrics

We use C-index, a widely used metric in survival analysis, to evaluate the model’s predictive accuracy by estimating the probability that the predicted outcomes align with the observed survival data. For any pair of patients, the prediction is considered concordant if the model assigns a higher risk score to the patient with a shorter observed survival time or a lower survival probability. Notably, pairs where one or both patients experienced the event (e.g. death) before the observation window were excluded from the calculation to avoid bias.


(10) $$C-\mathrm{index}=\mathrm{Prob}\{\beta^{T}z_{i}^{b}>\beta^{T}z_{j}^{b}|O_{j}>O_{i},\Delta_{i}=1\},$$

C-index range from 0 to 1. Higher values indicate better predictive performance, while lower values suggest poorer performance.

To further evaluate the feature extraction patterns of each expert module, we employed the Adjusted Rand Index (ARI) (Warrens *et al*. 2022) to quantify the agreement between the cluster assignments in the learned feature space and the true cancer-type labels. This analysis helps validate whether the representations captured by different experts reflect biologically meaningful cancer-type distinctions.

The ARI is calculated as follows:


(11) $$\mathrm{ARI}=\frac{\sum_{ij}C_{n_{ij}}^{2}-[\sum_{i}C_{a_{i}}^{2}\sum_{j}C_{b_{j}}^{2}]/C_{n}^{2}}{[\sum_{i}C_{a_{i}}^{2}+\sum_{j}C_{b_{j}}^{2}]/2\;-[\sum_{i}C_{a_{i}}^{2}\sum_{j}C_{b_{j}}^{2}]/C_{n}^{2}},$$

Where $n_{ij}$ denotes the number of samples commonly assigned to the *i*-th true cancer type and the *j*-th cluster in the feature space; $a_{i}$ is the total number of samples belonging to the *i*-th cancer type, and $b_{j}$ is the total number of samples assigned to the j-th cluster. The feature-space clustering was performed using the K-means algorithm, with the number of centers set to 18 to match the number of distinct cancer types. The ARI ranges from –1 to 1, where a value close to 1 indicates strong agreement between the two partitions.

## 4 Results

### 4.1 Evaluation of survival prediction performance in zero-shot cancer types

We evaluated the performance of MoESurv against several established survival prediction algorithms, including two classical supervised learning methods—DeepSurv (Katzman *et al*. 2018) and DeepHit (Lee *et al*. 2018), a meta-learning-based survival prediction algorithm (Qiu *et al*. 2020), and two variational autoencoder-based methods, VAECox (Kim *et al*. 2020) and AutoSurv (Jiang *et al*. 2024). To ensure a fair comparison, we set the hidden layer dimensions of DeepSurv and DeepHit to match those of MoESurv. Specifically, the original gene expression matrix is first linearly projected to a 2048-dimensional space, passed through a ReLU activation, and then linearly mapped to 1024 dimensions. For VAECox, the meta-learning model, and AutoSurv—which were originally designed for transcriptomic data—we adopted the hyperparameters recommended in their respective original publications and trained them on the 18 common cancer types. Consistent with the MoESurv, the prediction phase for these baselines involved first extracting embedding features from both the target cancer type and the training cancer types using their own encoders. A CoxPH model was then fitted on the embeddings from the training cancer types and finally perform the survival prediction on embeding of target cancer type. To ensure the robustness of the evaluation and prevent potential randomness, we employed the bootstrap resampling method (Efron 1987) to estimate the 95% confidence interval of the C-index predicted by the model. A lower bound greater than 0.5 indicates that the model’s predictive performance is significantly superior to random guessing.

As shown in Table 3, MoESurv demonstrated superior overall performance compared to the six baseline methods. It achieved the highest C-index of 0.6800 on the ACC cancer type and delivered strong results of 0.7406, 0.6973, and 0.7393 on KICH, MESO, and PCPG, respectively. Notably, on KICH, MoESurv outperformed the second-best method by a margin of 10 percentage points in C-index. MoESurv also attained the highest average C-index of 0.6645 across all rare cancer types, indicating significantly more robust and stable zero-shot prediction capability than the other baseline models.

**Table 3:** Predictive performance (C-index [95% CI]) of six survival prediction algorithms in zero-sample scenarios on rare/small-sample cancer types.

Method	ACC	CHOL	READ	KICH	MESO	PCPG	UCS	Average
DeepSurv	0.4659 [0.3369,0.6064]	0.4361 [0.2955,0.5651]	0.6056 [0.4307,0.7516]	0.5815 [0.3912,0.7544]	0.6175 [0.5402,0.6929]	0.6507 [0.4534,0.8484]	0.6278 [0.4956,0.7379]	0.5677
DeepHit	0.6531 [0.5348,0.7569]	0.3730 [0.2492,0.5560]	0.5619 [0.4136,0.7105]	0.6389 [0.4579,0.7981]	0.6600 [0.5780,0.7311]	0.6619 [0.3619,0.8434]	0.5234 [0.4108,0.6290]	0.5817
VAECox	0.6713 [0.5433,0.7986]	**0.5902 [0.4549,0.7142]**	0.6145 [0.4610,0.7622]	0.5554 [0.3890,0.7379]	0.6470 [0.5668,0.7235]	0.5866 [0.3972,0.8065]	0.5420 [0.4154,0.6437]	0.6010
Meta Learning	0.5682 [0.4214,0.7019]	0.5447 [0.4009,0.6792]	**0.6343 [0.4413,0.7639]**	0.6845 [0.4722,0.8229]	0.6937 [0.6182,0.7526]	0.6752 [0.3565,0.9147]	0.5650 [0.4724,0.6717]	0.6237
AutoSurv	0.6168 [0.4863,0.7344]	0.5482 [0.4071,0.6829]	0.5183 [0.3814,0.6574]	0.6441 [0.4220,0.7944]	0.4944 [0.4068,0.5760]	**0.7862 [0.5183,0.9604]**	0.4094 [0.3038,0.5281]	0.5739
MoESurv	**0.6800 [0.5443,0.7900]**	0.5412 [0.3718,0.6788]	0.6234 [0.4322,0.7605]	**0.7406 [0.5577,0.8656]**	**0.6973 [0.6216,0.7554]**	0.7393 [0.4929,0.9414]	**0.6295 [0.5330,0.7247]**	**0.6645**

Bold text indicates the highest metric among the methods.

**Figure 2:**
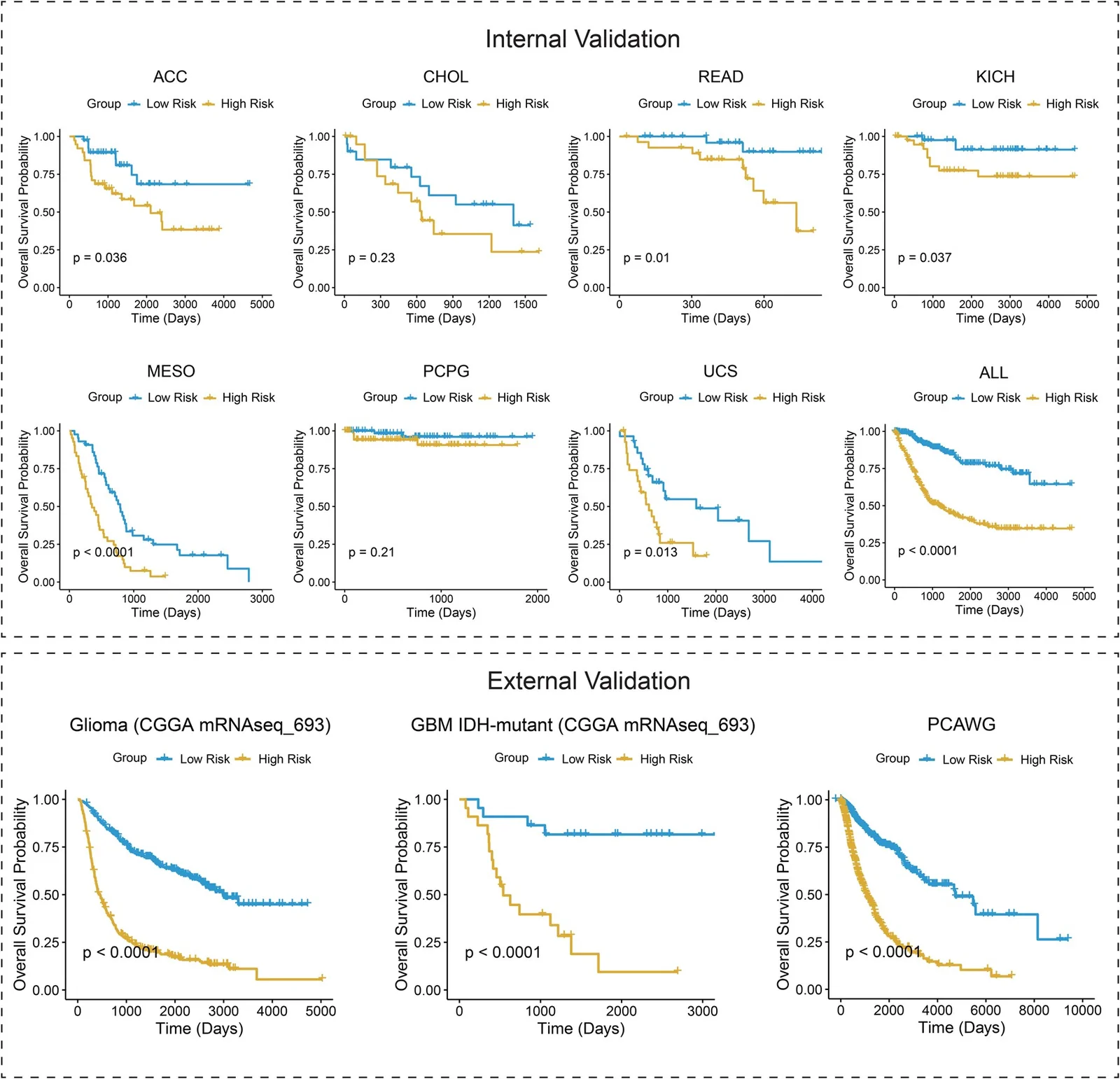
Kaplan–Meier survival curves stratified by MoESurv-predicted risk scores. Patients were divided into high—and low—risk groups based on the median risk score predicted by MoESurv. Kaplan—Meier curves are presented for each of the seven cancer types (ACC, CHOL, READ, KICH, MESO, PCPG, UCS) in the internal validation cohort, as well as for the combined cohort of all seven cancers. In addition, Kaplan—Meier curves for external validation cohorts (CGGA mRNAseq_693, GBM IDH—mutant subset of CGGA mRNAseq_693, PCAWG) are also shown. Log—rank test P—values are provided to indicate the significance of survival differences between the high—and low—risk groups in each cohort.

Figure 2 presents Kaplan-Meier survival curves for high- and low-risk groups stratified by MoESurv’s predicted risks. The “ALL” curve represents the composite prediction of all seven rare cancer types directly based on the Cox-PH model trained with shared-embedding of training data. For five of the seven rare cancers, as well as the overall seven cancers, the high-risk group showed significantly lower survival rates than the low-risk group within the corresponding time intervals. The *P*-values were less than 0.0001 for MESO and the overall prediction, 0.01 for READ, and below 0.05 for ACC and KICH. Nevertheless, the high-risk curve for PCPG still trended lower than the low-risk curve. These results collectively indicate that MoESurv effectively distinguishes patient groups with different prognostic risks and exhibits strong zero-shot prediction performance across multiple rare cancer types.

In external validation, MoESurv also demonstrated the most robust predictive performance among the small-sample algorithms (Table 4). In all external validation cohorts, Kaplan-Meier curves revealed statistically significant survival differences between the high- and low-risk groups (Fig. 2). These external validation results indicate that MoESurv possesses excellent cross-ethnic and cross-study survival prediction potential, facilitating knowledge transfer across diverse patient cohorts.

**Table 4:** Predictive performance (C-index [95% CI]) of four algorithms designed for small-sample prediction on external validation datasets.

Method	Glioma (CGGA mRNAseq_693)	GBM IDH-mutant (CGGA mRNAseq_693)	PCAWG	Average
VAECox	0.6108 [0.5780, 0.6384]	0.7307 [0.6018, 0.8215]	0.3943 [0.3606, 0.4249]	0.5786
Meta Learning	0.7072 [0.6793, 0.7321]	0.3994 [0.2799, 0.5501]	0.5955 [0.5631, 0.6293]	0.5674
AutoSurv	0.4817 [0.4500, 0.5113]	0.6687 [0.5110, 0.7834]	0.5030 [0.5110, 0.7834]	0.5511
MoESurv	**0.7433 [0.7161, 0.7643]**	**0.8064 [0.6817, 0.8871]**	**0.7090 [0.6817, 0.8871]**	**0.7529**

Bold text indicates the highest metric among the methods.

### 4.2 Analysis of different expert modules

**Figure 3:**
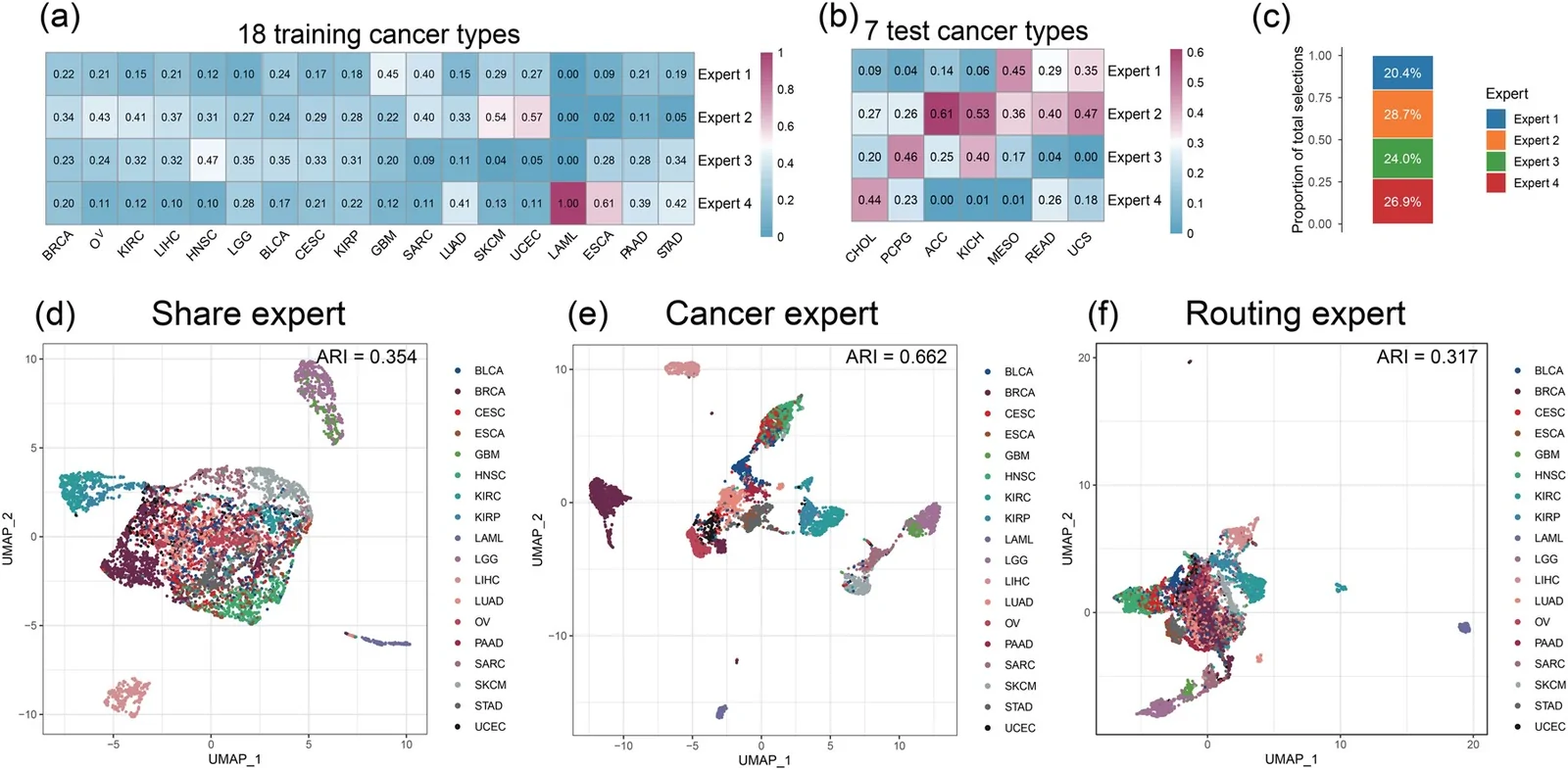
Feature extraction patterns of the three expert modules. (a) Heatmap showing the selection probability distribution of the four routing experts across the 18 cancer types in the training set. (b) Heatmap showing the selection probability distribution of the four routing experts across the 7 cancer types in the test set. (c) Overall selection probability of each routing expert within the training set. (d) Distribution of features extracted by the shared experts from gene expression data of the 18 training cancers, visualized after dimensionality reduction using UMAP. The Adjusted Rand Index (ARI) evaluates the agreement between the true cancer types and the clustering results obtained in the feature space. (e) Distribution of features extracted by the cancer-type experts from gene expression data of the 18 training cancers. (f) Distribution of features extracted by the routing experts from gene expression data of the 18 training cancers.

To further investigate the feature extraction preferences of the routing experts and validate the balance in their usage, we analyzed the probability of each routing expert being selected across different cancer types. As shown in Fig. 3, routing expert 1 was selected with high probability in GBM and SARC samples (0.45 and 0.40, respectively), indicating a preference for mesenchymal-origin tumors, which aligns with its frequent selection for MESO samples in the test set. Routing expert 2 showed a tendency to be selected for OV, KIRC, SKCM, and UCEC samples (probabilities of 0.43, 0.41, 0.54, and 0.57), reflecting a focus on tumors of the reproductive system, kidney, and skin, consistent with its selection pattern for KICH and UCS during testing. Routing expert 3 was predominantly chosen for HNSC samples (probability 0.47). Routing expert 4 was frequently activated for LUAD, LAML, ESCA, and STAD samples (probabilities of 0.41, 1.00, 0.61, and 0.42), suggesting a specialization in epithelial cancers of the digestive and respiratory systems, which corresponds to its selection for CHOL samples in the test set. In the test set, the selection probabilities of the experts also exhibited clear distinctions across different rare cancer types. Although the selection distributions varied considerably among experts, the overall probability of each expert being selected across all training samples remained near 0.25 (Fig. 3c), indicating that no single expert was overly dominant and confirming the effectiveness of the load-balancing loss.

Furthermore, we performed dimensionality reduction on the features extracted by each expert type from all training samples. As visualized in Fig. 3d–f, the features from the shared experts appeared highly intermixed in the low-dimensional space (ARI = 0.354), with sample points from different cancer types extensively overlapping, suggesting that these experts capture fundamental characteristics common across cancer types. In contrast, the features from the cancer-type experts demonstrated clear intra-class cohesion and inter-class separation between cancer types (ARI = 0.662), indicating successful learning of cancer type-specific patterns. The observed inter-type blending (ARI = 0.317) in the features from the routing experts suggests their effectiveness in capturing variations beyond the primary cancer types, which aligns with their role in adaptively extracting nuanced, task-relevant features. These visual results collectively demonstrate that the proposed expert modules work in a synergistic, complementary, and interpretable manner, which underlies the strong generalization capability achieved by the shared experts.

### 4.3 Identification of potential therapeutic targets in rare cancers

**Figure 4:**
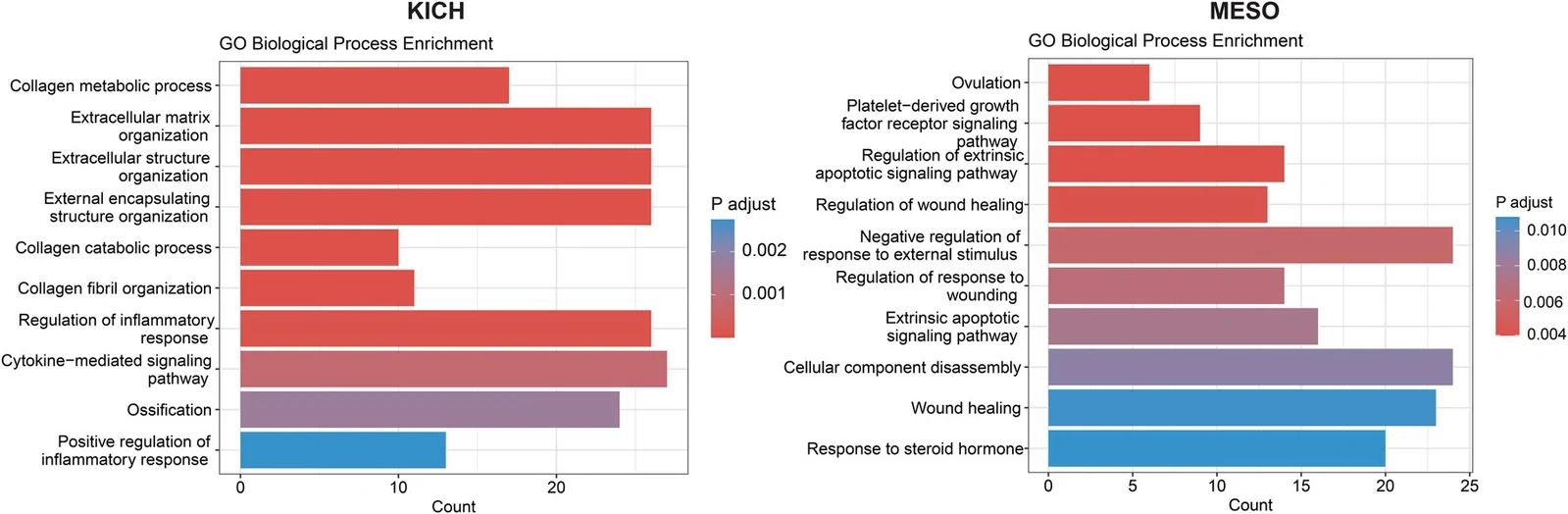
Pathway enrichment analysis between high- and low-risk groups defined by MoESurv. Differentially expressed genes between MoESurv-predicted high- and low-risk groups in KICH and MESO cancers were identified using the Wilcoxon rank-sum test (*P* < 0.05). Gene Ontology (GO) enrichment analysis was then performed using the top 500 most significant genes.

To clarify the biological relevance of the MoESurv model, we conducted an in-depth analysis of KICH and MESO, two cancer types for which MoESurv demonstrated superior predictive performance compared to conventional methods. Samples were stratified into high-risk and low-risk groups based on the median of the MoESurv-predicted risk scores. We then extracted genes that showed significant differential expression between the two groups. Given that gene expression data often do not follow a normal distribution and sample sizes are limited, the nonparametric Wilcoxon rank-sum test was employed to assess significance (*P* < 0.05). Subsequently, the top 500 most differentially expressed genes were selected for Gene Ontology (GO) enrichment analysis (Ashburner *et al*. 2000) to explore their underlying biological functions (Fig. 4).

As shown in Table 5, in KICH, upregulated genes in the high-risk group included SPHK1, MTHFD2, GFPT2, TXNRD1, and BEST1. GO analysis revealed significant enrichment of the differential genes in processes related to extracellular matrix (ECM) organization, collagen metabolism, cell adhesion, and regulation of inflammatory responses (Fig. 4). This is consistent with prior studies reporting that genes such as SPHK1, GFPT2, and MTHFD2 promote tumor progression in other cancers by modulating ECM remodeling, the immune microenvironment, and metabolic reprogramming (Zhang *et al*. 2010, 2023, Yue *et al*. 2022, Bao *et al*. 2023), suggesting that MoESurv has captured key malignant features associated with stromal remodeling and immunosuppression in KICH.

**Table 5:** Potential therapeutic drugs targeting differentially expressed genes in KICH and MESO.

Gene	*P* value	Regulation (High risk group)	Cancer	Approved drug	Unapproved drug
GFPT2	9.97E-06	Up	KICH	Not Found	Not Found
TXNRD1	1.38E-05	Up	KICH	Arsenic Trioxide, ASCORBIC ACID, AUROTHIOGLUCOSE	Motexafin Gadolinium, FOTEMUSTINE, SPERMIDINE
BEST1	3.48E-05	Up	KICH	Not Found	Not Found
SPHK1	3.63E-05	Up	KICH	Not Found	OPAGANIB, DIMETHYLSPINGOSINE, CHEMBL: CHEMBL554130, IDRONOXIL
MTHFD2	4.14E-05	Up	KICH	Not Found	Not Found
NBPF14	4.99E-05	Up	KICH	Not Found	Not Found
YWHAZP3	6.11E-05	Up	KICH	Not Found	Not Found
OASL	6.63E-05	Up	KICH	RIBAVIRIN, PEGINTERFERON ALFA-2B,	Not Found
ULBP2	6.98E-05	Up	KICH	Not Found	Not Found
COL5A1	7.94E-05	Up	KICH	COLLAGENASE CLOSTRIDIUM HISTOLYTICUM, OCRIPLASMIN	Not Found
MIPOL1	8.11E-05	Up	KICH	Not Found	Not Found
STEAP1	8.61E-05	Up	KICH	Not Found	VANDORTUZUMAB VEDOTIN
ATP6V1G1	3.94E-06	Down	MESO	Not Found	Not Found
KCNK1	5.21E-05	Down	MESO	Not Found	Not Found
POLE3	6.74E-05	Down	MESO	Gemcitabine, Clofarabine, Cytarabine, Fludarabine	Troxacitabine, Aspacytarabine
ERRFI1	0.000102	Down	MESO	Not Found	EPIDERMAL GROWTH FACTOR RECEPTOR TYROSINE KINASE INHIBITOR
PLK2	0.000118	Down	MESO	Not Found	Wortmannine, BI-2536
CREB5	0.000142	Down	MESO	Not Found	Not Found
NEDD4L	0.000152	Down	MESO	AMILORIDE, CITALOPRAM	DIURETIC
KDM6B	0.000182	Down	MESO	Not Found	Not Found
MSANTD3	0.000187	Down	MESO	Not Found	Not Found
ZFAND5	0.00021	Down	MESO	Not Found	Not Found
TIGAR	0.00023	Down	MESO	Not Found	Not Found

For MESO, the analysis identified genes with marked expression differences, including ATP6V1G1, KCNK1, PLK2, CREB5, and POLE3. GO enrichment pointed to pathways involved in cellular stress responses, regulation of apoptotic processes, and ion homeostasis (Fig. 4). Among these, KCNK1, CREB5, and ATP6V1G1 have been implicated in proliferation, metastasis, and metabolic reprogramming in pan-cancer studies (Contreras-Sanzón *et al*. 2022, Sun *et al*. 2023, Zhang *et al*. 2024), though the specific roles of some genes, such as PLK2, remain less defined in MESO. The significant upregulation of ROS-related genes in the high-risk group further suggests that oxidative stress may play an important role in MESO progression.

To further validate the clinical relevance of these findings, we performed a drug-target analysis on the differentially expressed genes based on DGIdb database (Cannon *et al*. 2024) (Table 5), which is a comprehensive resource that aggregates drug–gene interaction information from multiple sources, providing evidence-based and manually curated data to facilitate the identification of potential therapeutic agents and drug repurposing opportunities. For KICH, the upregulated genes GFPT2, TXNRD1, and SPHK1 were found to be potential targets for approved drugs such as Arsenic Trioxide, Ascorbic Acid, and Motexafin Gadolinium, suggesting existing therapeutic avenues for high-risk patients. In MESO, genes like POLE3 and ERRFI1 showed potential sensitivity to drugs including Troxacitabine and EGFR inhibitors, respectively. This analysis not only corroborates the biological significance of the identified genes but also highlights the potential for repurposing existing drugs or developing targeted therapies based on the MoESurv-derived risk stratification.

Notably, both the highly differential genes and the enriched pathways pointed to relatively conserved biological processes closely linked to disease progression and prognosis. Many of these genes and pathways have not been systematically studied or reported in the context of KICH and MESO. This indicates that the shared experts in MoESurv successfully extracted more generalizable survival-related features from pan-cancer data, rather than overfitting to cancer-type-specific signals. This finding provides a biological interpretation for the robust generalization capability of the MoESurv model.

### 4.4 Ablation study

To comprehensively evaluate the contribution of each component in MoESurv, we conducted ablation studies on two distinct rare cancer types, ACC and PCPG, examining six key design aspects. Results are summarized in Table 6, with performance measured by the C-index on ACC, PCPG, and their average.

**Table 6:** Ablation study with C-index [95% CI] in ACC and PCPG.

Ablation method	ACC	PCPG	Average
**Experiment 1: Validating the Effectiveness of the Generalizable Supervised Training Method** (Configuration: 4 routing experts, 1 cancer-type expert per cancer, 1 shared expert; loss weights—risk: 1, reconstruction: 1, balance: 0.01)			
Optimize both the reconstruction loss and the risk prediction loss within the same batch of data.	0.5073 [0.3820,0.6295]	0.6497 [0.3259,0.9010]	0.5785
Optimize only the reconstruction loss without considering survival risk.	**0.6901 [0.5799,0.7838]**	0.4908 [0.1365,0.8902]	0.5905
Optimize only the survival risk without optimizing the reconstruction loss.	0.5602 [0.4586,0.6778]	0.5713 [0.1774,0.9146]	0.5658
Use the proposed generalizable supervised training method (our method).	0.6800 [0.5443,0.7900]	**0.7393 [0.4929,0.9414]**	**0.7097**
**Experiment 2: Validating the Effectiveness of the Balance Loss** (Configuration: 4 routing experts, 1 cancer-type expert per cancer, 1 shared expert; loss weights—risk: 1, reconstruction: 1; using the generalizable supervised training method)			
Balance weight: 0.	0.6524 [0.5120,0.7704]	0.6477 [0.4346, 0.8298]	0.6501
Balance weight: 0.01 (our method).	**0.6800[0.5443,0.7900]**	**0.7393[0.4929,0.9414]**	**0.7097**
**Experiment 3: Comparing the Suitability of Autoencoders and Variational Autoencoders** (Configuration: 4 routing experts, 1 cancer-type expert per cancer, 1 shared expert; loss weights—risk: 1, reconstruction: 1, balance: 0.01; using the generalizable supervised training method)			
Using a variational Autoencoder as the feature extraction network.	0.5820 [0.4506,0.7083]	0.6873 [0.3753,0.9417]	0.6347
Using an Autoencoder as the feature extraction network (our method).	**0.6800 [0.5443,0.7900]**	**0.7393 [0.4929,0.9414]**	**0.7097**
**Experiment 4: Evaluating the Composition of Expert Modules** (Configuration: loss weights—risk: 1, reconstruction: 1, balance: 0.01; using the generalizable supervised training method)			
1 shared experts + 1 cancer-type expert per cancer.	0.5856 [0.4476,0.7100]	**0.7393 [0.4723,0.9476]**	0.6625
1 shared experts + 4 routing experts.	0.6393 [0.5214,0.7656]	0.7257 [0.3835,0.8887]	0.6825
1 shared experts + 4 routing experts + 1 cancer-type expert per cancer (our method).	**0.6800 [0.5443,0.7900]**	**0.7393 [0.4929,0.9414]**	**0.7097**
**Experiment 5: Investigating the Impact of the Number of Routing Experts on Model Performance** (Configuration: 1 cancer-type expert per cancer, 1 shared expert; loss weights—risk: 1, reconstruction: 1, balance: 0.01; using the generalizable supervised training method)			
Number of routing experts: 2	**0.7338 [0.6207,0.8129]**	0.5509 [0.3397,0.8107]	0.6424
Number of routing experts: 4	0.6800 [0.5443,0.7900]	**0.7393 [0.4929,0.9414]**	**0.7097**
Number of routing experts: 6	0.6118 [0.4566, 0.7296]	0.7016 [0.4309,0.9222]	0.6567
**Experiment 6: Comparing the Effectiveness of MoESurv Against a cycle Generative Adversarial Network (cycle-GAN)**			
Cycle-GAN	**0.6909 [0.5686,0.7744]**	0.3839 [0.2169, 0.5302]	0.5374
Our method	0.6800 [0.5443,0.7900]	**0.7393 [0.4929,0.9414]**	**0.7097**

Bold text indicates the highest metric among the methods.

We first compared different training strategies while keeping other modules fixed. Jointly optimizing the reconstruction and risk losses within the same batch yielded an average C-index of only 0.5785, indicating poor synergy. Optimizing only the reconstruction loss resulted in high performance on ACC (0.6901) but a significant drop on PCPG (0.4908). Using only the risk loss led to balanced but overall modest performance (average 0.5658). In contrast, the proposed generalizable supervised training paradigm achieved the best overall results (ACC = 0.6800, PCPG = 0.7393, average = 0.7097), demonstrating its effectiveness in enhancing model generalizability.

The importance of the balance loss was assessed by adjusting its weight. Setting the weight to zero (i.e. removing the loss) reduced the average performance from 0.7097 to 0.6501 (ACC = 0.6524, PCPG = 0.6477), confirming that the balance loss is essential for the proper functioning of the mixture-of-experts system.

We also compared using an autoencoder (AE) versus a variational autoencoder (VAE) as the feature extraction network. The VAE achieved an average C-index of 0.6347, with higher performance on PCPG but lower on ACC. The standard AE (the proposed method) delivered superior and more stable overall performance (average 0.7097), suggesting its greater representational stability for this task.

Different expert combinations were evaluated. Using only shared and cancer-type experts yielded an average C-index of 0.6625, with reasonable PCPG performance but low ACC. Using only shared and routing experts improved the average to 0.6825. The full model incorporating all three expert types achieved the best performance (average 0.7097), indicating complementary and synergistic roles where the shared experts can focus on learning generalizable features.

Varying the number of routing experts ($n_{r}$=2,4,6) revealed that $n_{r}$=2 led to the highest ACC but significantly lower PCPG, indicating underfitting. $n_{r}$=6, ACC decreased noticeably. Setting $n_{r}$=4 produced the optimal balance (average 0.7097), demonstrating that an appropriate number of routing experts is crucial for stable multi-task generalization.

Regarding cycle-GAN (Generative Adversarial Network) (Zhu *et al*. 2017) also possess the ability to extract commonalities among individuals across different categories, we compared MoESurv against a model using a cycle-GAN with an encoder having the same architecture as the shared expert for feature extraction. While the GAN-based model performed well on ACC (0.7078), its performance on PCPG was substantially lower (0.3574), resulting in a much lower average C-index of 0.5326 compared to MoESurv’s 0.7097. This confirms that the multi-expert mechanism in MoESurv is more effective at extracting generalizable, survival-related features for risk prediction across different cancers.

## 5 Discusion and conclusion

In this study, we developed MoESurv, a novel zero-shot survival prediction framework designed to bypass the severe data-scarcity bottleneck inherent in rare cancers. Unlike conventional transfer learning or meta-learning paradigms that heavily rely on target-domain fine-tuning—a luxury seldom afforded by ultra-rare malignancies—MoESurv achieves robust pan-cancer generalization without any prior exposure to the target domain.

The architectural synergy between the Autoencoder (AE) and the Mixture of Experts (MoE) is central to this capability. By decoupling patient representations into three types of expert system, MoESurv successfully mitigates the risk of negative transfer. The routing network effectively isolates domain-specific clinical noise and batch effects, allowing the shared experts to focus purely on extracting fundamental, cross-cancer prognostic signals. Consequently, the model demonstrates superior robustness and stability across highly heterogeneous external validation cohorts.

Beyond its methodological novelty, MoESurv offers profound biological insights into the conserved molecular landscapes of rare tumors. Our finding that models trained on common cancers can successfully stratify risks in rare cohorts (e.g. KICH and MESO) validates a core scientific hypothesis: despite distinct anatomical origins, rare and common malignancies often converge on shared downstream oncogenic programs, such as immune evasion or metabolic reprogramming. Specifically, the identification of SPHK1 and TXNRD1 as pivotal prognostic drivers bridges the gap between statistical correlation and biological plausibility, as upregulation of SPHK1 and TXNRD1 is widely documented to promote aggressive tumor progression. By pinpointing these conserved features, MoESurv facilitates a rational framework for drug repurposing (e.g. targeting high-risk groups with arsenic trioxide or ascorbic acid). This provides an actionable, low-cost pipeline for precision oncology in orphan diseases where large-scale clinical trials are notoriously difficult to execute.

Despite these promising capabilities, several limitations warrant acknowledgment. First, our current model relies primarily on transcriptomic profiles, omitting the rich spatial and structural information embedded in digital whole-slide images (WSIs) or radiology data. Integrating multi-modal architectures remains a critical avenue for future work. Second, while the model demonstrates exceptional mathematical consistency, the identified drug-target interactions remain predictive hypotheses. Future studies will focus on validating these candidate therapies using patient-derived organoids or in vivo models to fully bridge the gap between AI prediction and clinical application.

## Author contributions

Shuping Fang (Conceptualization [Lead], Data curation [Lead], Formal analysis [Lead], Investigation [Lead], Methodology [Lead], Project administration [Lead], Software [Lead], Validation [Lead], Writing—original draft [Lead], Writing—review & editing [Lead]), Yuhang Wang (Conceptualization [Lead], Data curation [Lead], Formal analysis [Lead], Investigation [Lead], Methodology [Lead], Visualization [Lead], Writing—original draft [Lead], Writing—review & editing [Lead]), Mengyan Zhou (Validation [Equal], Visualization [Equal]), Yanting Shao (Investigation [Equal]), Zhenghao Jiang (Investigation [Equal]), Yitong Guo (Investigation [Equal]), Tao Jiang (Investigation [Equal]), Feng Wang (Resources [Lead], Supervision [Lead]), Jingya Fang (Resources [Lead], Supervision [Lead]), and Hong Tian (Conceptualization [Lead], Formal analysis [Lead], Project administration [Lead], Resources [Lead], Supervision [Lead])

## Supplementary Material

btag549_Supplementary_Data

## Data Availability

All data used in this manuscript are publicly available. The TCGA dataset used in this work can be obtained from the website https://www.cancer.gov/ccg/research/genome-sequencing/tcga. The PCAWG dataset is available on the website http://xena.ucsc.edu/. The CGGA dataset is available on the website https://www.cgga.org.cn/.
