# Supplementary material for: MoESurv: a zero-sample and transferable survival prediction framework for rare cancers using mixture of experts
Source: Bioinformatics. 2026 Jul 22;42(8):btag549. doi: 10.1093/bioinformatics/btag549 (PMC13472725; doi:10.1093/bioinformatics/btag549)
Supplement: Supplementary file 1 [file btag549_supplementary_data.docx]

**Figure S1：Training curves of the total loss, risk prediction loss, reconstruction loss, and balance loss.**

**Table S1. DeepSurv and meta learning algorithms were trained using shared expert features derived from MoESurv. The figure presents the C-index along with its 95% confidence intervals. The green text indicate performance improvements achieved by substituting gene expression profiles with MoESurv embeddings as input.**

| **Method** | **ACC** | **CHOL** | **READ** | **KICH** | **MESO** | **PCPG** | **UCS** | **Average** |
| --- | --- | --- | --- | --- | --- | --- | --- | --- |
| DeepSurv | 0.4659  [0.3369,0.6064] | 0.4361  [0.2955,0.5651] | 0.6056  [0.4307,0.7516] | 0.5815  [0.3912,0.7544] | 0.6175  [0.5402,0.6929] | 0.6507  [0.4534,0.8484] | 0.6278  [0.4956,0.7379] | 0.5677 |
| DeepSurv with MoE Embedding | 0.6447  [0.5313,0.7756] | 0.5131  [0.3512,0.6419] | 0.6095  [0.4333,0.7574] | 0.6897  [0.4750,0.8250] | 0.6988  [0.6210,0.7596] | 0.6711  [0.3689,0.9342] | 0.6083  [0.5029,0.6938] | 0.6336 |
| Meta Learning | 0.5682  [0.4214,0.7019] | **0.5447**  **[0.4009,0.6792]** | **0.6343**  **[0.4413,0.7639]** | 0.6845  [0.4722,0.8229] | 0.6937  [0.6182,0.7526] | 0.6752  [0.3565,0.9147] | 0.5650  [0.4724,0.6717] | 0.6237 |
| Meta Learning with MoE Embedding | 0.6611  [0.5313,0.7765] | 0.5342  [0.3778,0.6764] | 0.6105  [0.4394,0.7585] | 0.7210  [0.4919,0.8647] | 0.6918  [0.6096,0.7518] | 0.7108  [0.4231,0.9326] | 0.5968  [0.4973,0.6772] | 0.6466 |
| MoESurv | **0.6800**  **[0.5443,0.7900]** | 0.5412  [0.3718,0.6788] | 0.6234  [0.4322,0.7605] | **0.7406**  **[0.5577,0.8656]** | **0.6973**  **[0.6216,0.7554]** | 0.7393  [0.4929,0.9414] | **0.6295**  **[0.5330,0.7247]** | **0.6645** |

**Table S2. Hyperparameter settings for MoESurv**

| Hyper-Parameters | Our settings |
| --- | --- |
| Training iteration | 3000 |
| Optimizer | ADAM |
| Dimension of latent space | [2048,1024] |
| Activation function | ReLU |
| Umsampling rate before ReLU | 2 |
| Batch size for risk prediction | 256 |
| Batch size for reconstruction prediction | 512 |
| Number of shared expert | 1 |
| Number of routing expert | 4 |
| Number of cancer expert | 1 per cancer type of training set |
| Learning rate | 1e-5 |
| Weight of balance loss | 0.01 |
| Weight of risk loss | 1 |
| Weight of reconstruction loss | 1 |
| Hyper-Parameters | Our settings |

**Table S3. Clinical development status of candidate drugs targeting MoESurv-identified genes in rare diseases and specific rare cancers (KICH and MESO).**

| **Gene** | **Cancer** | **Approved drug** | **Status: for rare cancer treatment** | **Unapproved drug** | **Status: for rare cancer treatment** |
| --- | --- | --- | --- | --- | --- |
| TXNRD1 | KICH | Arsenic Trioxide | Phase 2: Acute Promyelocytic Leukaemia， APL NCT04687176，Recruiting | Motexafin Gadolinium | NA |
|  |  | ASCORBIC ACID | Phase3 :Charcot-Marie-Tooth Disease Type 1A, CMT1A, NCT00484510, completed | FOTEMUSTINE | NA |
|  |  | AUROTHIOGLUCOSE | NA | SPERMIDINE | NA |
| SPHK1 | KICH | Not Found | NA | OPAGANIB | NA |
|  |  |  |  | DIMETHYL SPINGOSINE | NA |
|  |  |  |  | CHEMBL:CHEMBL554130 | NA |
|  |  |  |  | IDRONOXIL | Phase 3:Fallopian Tube Cancer, NCT00382811, completed |
| OASL | KICH | RIBAVIRIN | Phase 2: Acute Myelogenous Leukemia, NCT02109744, completed | Not Found | NA |
|  |  | PEGINTERFERON ALFA-2B | Launched, Polycythemia Vera;  Phase 2: Craniopharyngioma, NCT01964300, Terminated |  |  |
| COL5A1 | KICH | COLLAGENASE | Lunched, Peyronie's Disease;  Phase1: Refractory Iatrogenic Esophageal Strictures, NCT03898661, Terminated. | Not Found | NA |
|  |  | CLOSTRIDIUM | NA |  |  |
|  |  | HISTOLYTICUM | Lunched, Peyronie's Disease |  |  |
|  |  | OCRIPLASMIN | Lunched, Vitreomacular Adhesion/ Vitreomacular Traction |  |  |
| STEAP1 | KICH | Not Found | NA | VANDORTUZUMAB VEDOTIN | NA |
| POLE3 | MESO | Gemcitabine | Phase3 : Malignant Pleural Mesothelioma, NCT03710876, Completed | Troxacitabine | Phase 2: Relapsed or Refractory Acute Myeloid Leukemia, NCT00129948, Completed |
|  |  | Clofarabine | Lunched, Pediatric Relapsed/Refractory ALL. |  |  |
|  |  | Cytarabine | Lunched, High-Risk AML. | Aspacytarabine | Phase 2: Relapsed or Refractory Acute Myeloid Leukemia, NCT02544438, Completed |
|  |  | Fludarabine | Lunched, B-cell Chronic Lymphocytic Leukemia, B-CLL |  |  |
| ERRFI1 | MESO | Not Found | NA | EPIDERMAL GROWTH FACTOR | NA |
|  |  |  |  | RECEPTOR TYROSINE KINASE INHIBITOR | Receptor Tyrosine Kinase Inhibitors” do not refer to a single specific drug, but rather to a broad class of medications (a category of molecularly targeted therapies).  As a whole, this class of drugs has not only undergone extensive clinical research for rare diseases, but a significant number of individual drugs within it have also successfully received approvals for rare disease (orphan drug) indications. |
| PLK2 | MESO | Not Found | NA | Wortmannine | NA |
|  |  |  |  | BI-2536 | Phase 2: Refractory or Relapsed Acute Myeloid Leukaemia, NCT00701766, Completed. |
| NEDD4L | MESO | AMILORIDE | Phase 2, Multiple Sclerosis, NCT01910259, Completed. | DIURETIC | NA |
|  |  | CITALOPRAM | Phase 2, Huntington's Disease, NCT00271596, Completed. |  |  |
